# Primary central nervous system T cell lymphoma involving multiple brain and spinal cord lesions with *SETD2* mutation

**DOI:** 10.1007/s12185-026-04262-7

**Published:** 2026-08-04

**Authors:** Ken Naganuma, Takeaki Matsunaga, Yuta Arai, Kumi Hirata, Taisuke Kawada, Noriyuki Sakata, Yasuyuki Takahashi, Masahiro Kizaki, Chiaki Murakami, Hiroki Imada, Takahisa Yamashita, Shuji Momose, Morihiro Higashi, Takayuki Tabayashi

**Affiliations:** 1 Department of Hematology, Saitama Medical Center, Saitama Medical University, Saitama, Japan; 2 Department of Pathology, Saitama Medical Center, Saitama Medical University, Saitama, Japan

**Keywords:** Primary central nervous system lymphoma, T cell lymphoma, Spinal cord involvement

## Abstract

Primary central nervous system lymphoma (PCNSL) is a rare extranodal lymphoma, with diffuse large B cell lymphoma accounting for most cases. T cell variants are uncommon and associated with poor outcomes. We report a case of primary CNS T cell lymphoma with simultaneous brain and spinal cord involvement. A 37-year-old Japanese man presented with impaired consciousness, and brain magnetic resonance imaging revealed multiple contrast-enhancing parenchymal lesions. Histopathological examination of a brain biopsy specimen demonstrated medium- to large-sized atypical lymphoid cells, leading to a diagnosis of peripheral T cell lymphoma, not otherwise specified (PTCL-NOS). Comprehensive systemic evaluation showed no extracranial disease; however, contrast-enhanced spinal magnetic resonance imaging revealed extensive lesions from the cervical spinal cord to the thoracic spinal cord. Next-generation sequencing analysis identified a mutation in SETD2, possibly contributing to the aggressive disease behavior. The patient received methotrexate, procarbazine, and vincristine as first-line therapy and achieved an initial radiological complete response. However, rapid disease relapse occurred with newly developed lesions in the pineal gland and cranial nerves, and the patient died shortly after initiation of consolidation therapy. This case highlights the aggressive clinical course of primary CNS T cell lymphoma with spinal cord involvement and underscores the importance of molecular genetic profiling in this rare entity.

**Supplementary Information:**

The online version contains supplementary material available at https://doi.org/10.1007/s12185-026-04262-7.

## Introduction

Primary central nervous system lymphoma (PCNSL) is a malignant extranodal lymphoma confined to the central nervous system, including the brain, spinal cord, cerebrospinal fluid, and eyes. Pathologically, the majority of cases are diffuse large B cell lymphoma (DLBCL), while T cell lymphoma is rare (2–4%) [1, 2]. These T cell variants are rare and generally associated with an aggressive clinical course and inferior survival compared with B cell PCNSL [3, 4]. Additionally, PCNSL typically presents as parenchymal brain lesions, while spinal cord involvement is exceedingly rare, occurring in less than 1% of cases [5]. Reports of primary CNS T cell lymphoma are particularly limited, and the clinicopathological and molecular characteristics of such cases remain poorly defined.

This case highlights the highly aggressive nature of primary CNS T cell lymphoma with spinal cord involvement and suggests that molecular genetic profiling may provide important insights into disease biology and therapeutic strategy in this rare entity.

### Case presentation

**Fig. 1:**
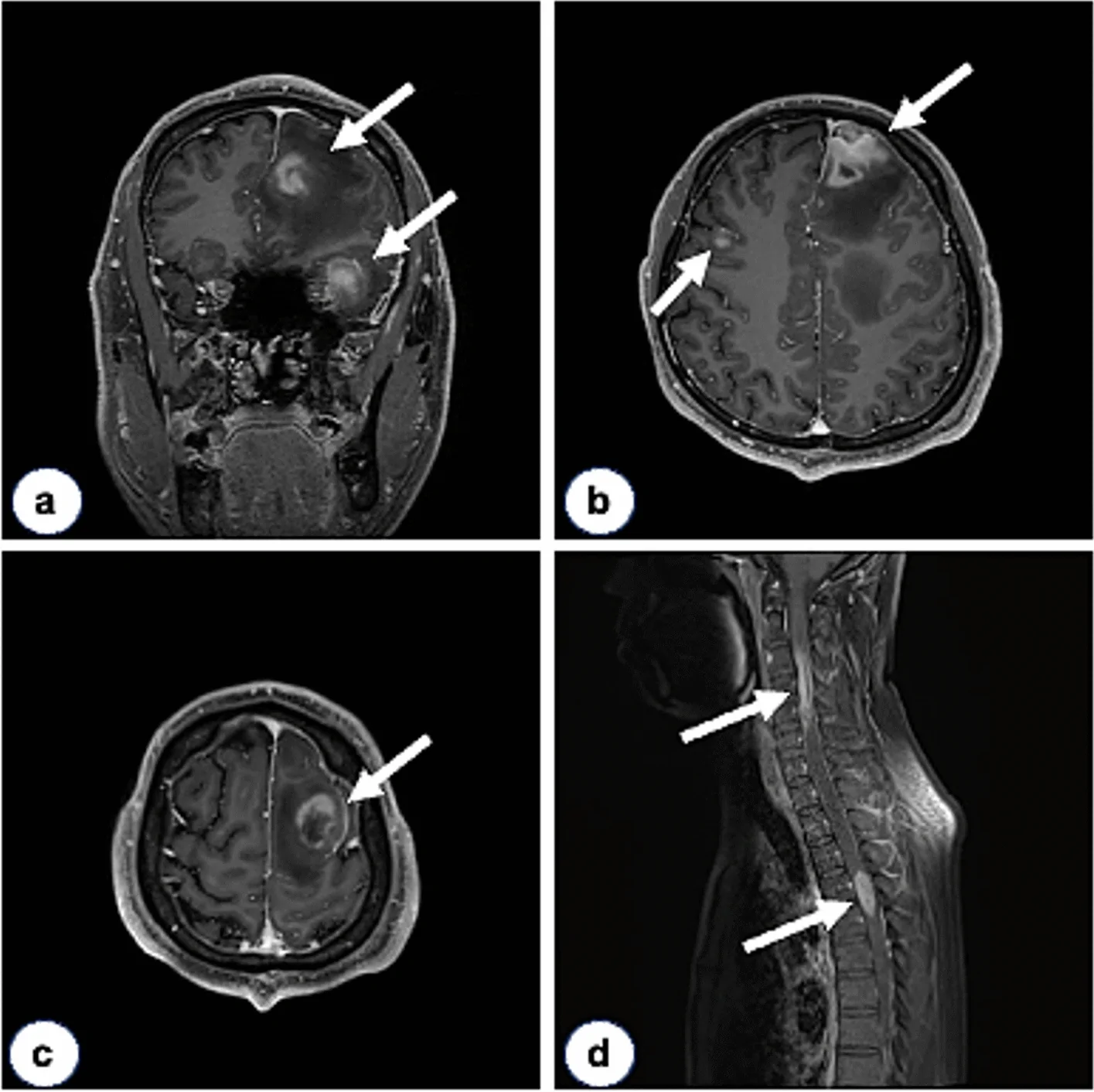
Images at diagnosis of primary diseases. **a, b, c** Contrast-enhanced magnetic resonance imaging (MRI) of the brain showing left frontal right frontal, left temporal, and left parietal lobe lesions (arrow). **d** Contrast-enhanced MRI of T2 high signal area spine at C 3/4/5 and Th 4/5 levels (arrow)

**Fig. 2:**
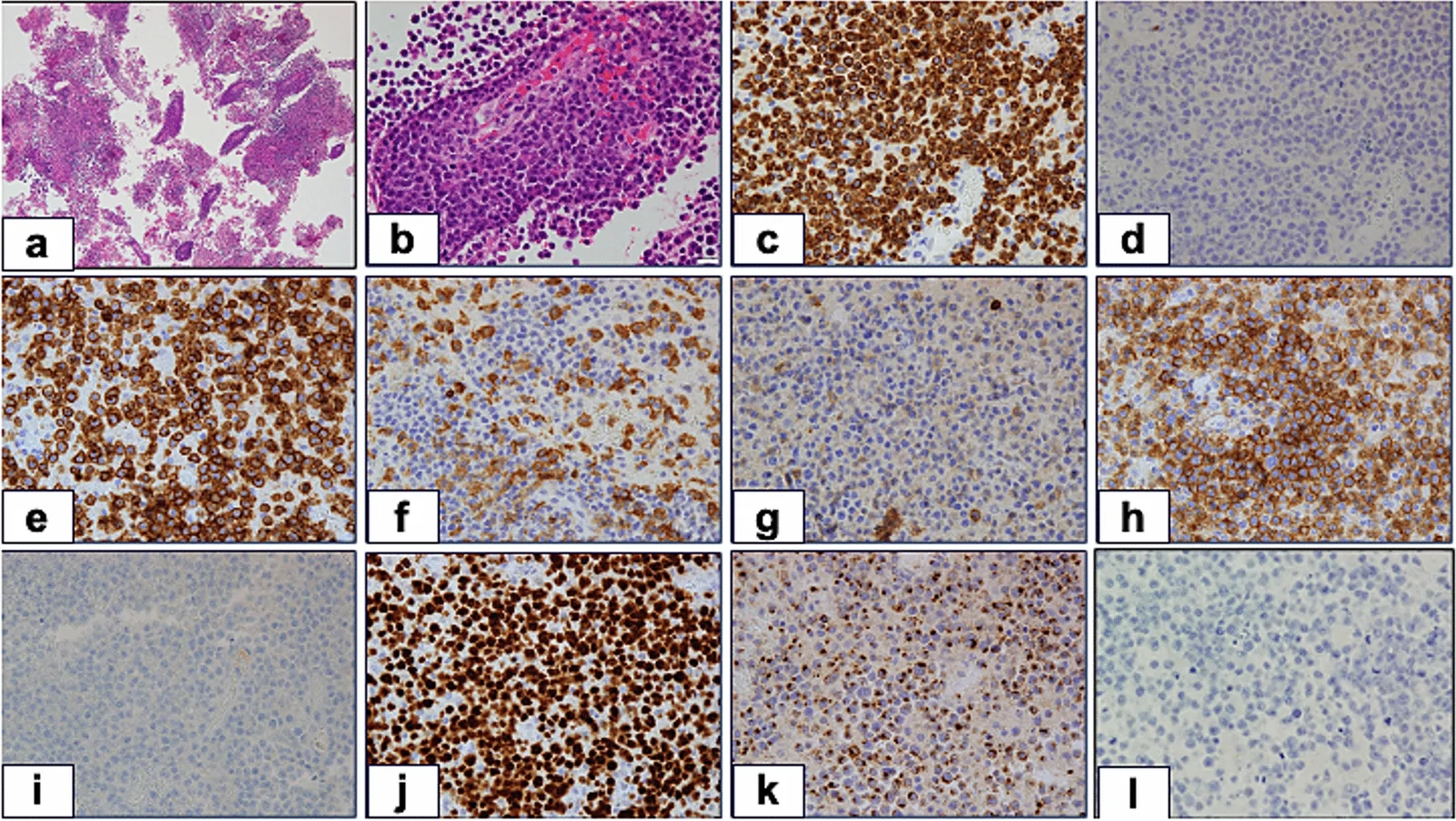
Histopathological findings at diagnosis of primary diseases of the biopsy from the frontal lesion. **a, b** Hematoxylin and eosin stained. Large lymphoid cells with nuclear indentations and clefts are proliferating perivascularly and within the brain parenchyma. **c** CD3. **d** CD20. **e** CD2. **f** CD4. **g** CD8. **h** CD30. **i** ALK. **j** Ki-67 with a proliferation index of 90%. **k** Granzyme B. **l** EBER-ISH

A 37-year-old Japanese man referred to our hospital complaining of disorders of consciousness. He had no significant past medical history. Cranial computed tomography (CT) and magnetic resonance imaging (MRI) showed multiple neoplastic lesions within the brain parenchyma. The lesions were as follows: (1) enhancing lesion in the left temporal lobe; (2) enhancing lesion in the left parietal lobe; (3) enhancing lesion in the right frontal lobe; and (4) enhancing lesion in the left frontal lobe (Fig. 1a–c). A brain biopsy was performed. The pathological findings showed medium- to large-sized lymphoid cells proliferating diffusely in the brain parenchyma. Immunohistochemical analysis revealed positivity for CD2, CD3, CD30, TIA-1, and granzyme B, with negativity for CD4, CD5, CD7, CD8, CD20, CD56, and ALK. Tumor cells were negative for in situ hybridization for Epstein–Barr virus-encoded RNA (EBER-ISH). No hallmark cells characteristic of anaplastic large cell lymphoma were found (Fig. 2). In addition, a subset of tumor cells exhibited weak or even negative CD30 staining. Based on both morphological and immunophenotypic findings, a pathological diagnosis of peripheral T cell lymphoma, not otherwise specified (PTCL-NOS), was made. Cervical-to-pelvic CT did not reveal malignant lymphoma lesions. The patient tested negative for HIV. Cytology of cerebrospinal fluid (CSF) was Class Ⅰ. Contrast-enhanced MRI scan performed to investigate postoperative quadriplegia revealed diffusely enlarged lesions from the cervical spinal cord to the thoracic spinal cord with contrast enhancement (Fig. 1d). Based on these findings, the patient was finally diagnosed with primary central nervous system peripheral T cell lymphoma with involvement of the brain parenchyma and spinal cord. To investigate the mutational status, next-generation sequencing (NGS) was performed on a biopsy sample using a targeted 130-gene panel developed by the Department of Pathology at Saitama Medical Center, Saitama Medical University [6] [7]. The PTCL-NOS cells had mutation in *SETD2*, with variant allele frequency (VAF) of 36.4% (Table 1). TCR clonality analysis was performed according to the BIOMED-2 protocol [8], and clonal bands of TCRβ and TCR*γ* were detected (Supplemental Fig. 1). He received five courses of methotrexate, procarbazine, and vincristine (MPV) as first-line treatment. One course of MPV therapy improved his loss of consciousness but not his quadriplegia. During the fourth course of MPV therapy, the patient developed septic shock due to *Pseudomonas aeruginosa*. The patient improved with antibiotic treatment, and the subsequent clinical course was uneventful. The best response to the MPV regimen was assessed as complete response (CR) based on contrast-enhanced brain MRI, which showed complete resolution of the previously identified lesions. Following the MPV regimen, he received high-dose cytarabine (HDAC) therapy as consolidation. However, the patient developed newly appearing disseminated lesions in the pineal gland and cranial nerves and died on day 17 of high-dose cytarabine therapy due to rapidly progressive CNS relapse involving the pineal gland and cranial nerves. Cervical-to-pelvic CT and spinal cord MRI at the time of recurrence showed no lymphoma lesions.

**Table 1:** Mutational status of genes

Gene	Amino acid change	Mutation	VAF (%)
SETD2	Pro914Ser	2716C > T	36.4

## Discussion

PCNSL is an uncommon form of extranodal non-Hodgkin lymphoma confined to the central nervous system, including the brain, spine, CSF and eyes, and the majority of its subtype is DLBCL [9]. PTCL accounts for 2–4% of PCNSL cases and is generally associated with an unfavorable prognosis [1–4].

The 5-year overall survival (OS) rates by subtype of PCNSL are follows: 30% for diffuse large B cell lymphoma, 66% for follicular lymphoma, 79% for marginal zone lymphoma, 42% for Burkitt lymphoma, 38% for small lymphocytic lymphoma, 45% for Hodgkin lymphoma, and 33% for PTCL, respectively. PTCL is associated with a poor prognosis [3]. Shenkier TN et al. reported a median progression-free survival (PFS) of 22 months and a median OS of 25 months with high-dose MTX-based therapy in a study of 45 patients with primary CNS T cell lymphoma [4].

Spinal cord involvement in PCNSL is exceedingly rare, occurring in less than 1% of cases, and has been associated with particularly poor outcomes [5]. Primary CNS T cell lymphoma with spinal cord involvement is extremely rare with limited reports [10–13]. Flanagan EP et al. reported that the 2-year OS rate for PCNSL with spinal cord lesions is 36%, indicating a poor prognosis [14].

High-dose methotrexate-based chemotherapy remains the cornerstone of PCNSL treatment [15]. MPV regimen has shown effectiveness [4,^16^], as demonstrated in our case where the patient experienced an improvement in consciousness after one course, although quadriplegia persisted. However, the patient developed septic shock during the fourth course of MPV therapy, which highlights the risk of severe infections during treatment. The best response achieved with the MPV regimen in our patient was CR. Craniospinal irradiation was not performed in this case because of concerns regarding treatment-related neurotoxicity, particularly given the extensive involvement of both cerebral and spinal lesions. Instead, consolidation with high-dose chemotherapy followed by autologous stem cell transplantation (ASCT) was planned after completion of high-dose cytarabine therapy, in accordance with contemporary treatment strategies for primary CNS T cell lymphoma in eligible patients. Despite achieving a CR with the MPV regimen, the patient’s relapse with disseminated lesions in the pineal gland and cranial nerves post-HDAC consolidation therapy reflects the aggressive relapse patterns. This underscores the necessity for novel therapeutic approaches and vigilant monitoring post-CR.

Recent molecular studies have identified recurrent mutations involving epigenetic regulators and the JAK/STAT signaling pathway in primary CNS T cell lymphoma [17, 18]. The tumor-suppressor role of *SETD2* is becoming increasingly recognized in several cancer types, including lymphoma [19]. Moffitt AB et al. demonstrated that loss-of-function alterations in *SETD2* are recurrent in monomorphic epitheliotropic intestinal T cell lymphoma and enteropathy-associated T cell lymphoma. These alterations are associated with impaired histone H3 lysine 36 trimethylation, genomic instability, and aggressive disease behavior. Given the essential role of *SETD2* in normal T cell development and genome maintenance, disruption of SETD2-mediated epigenetic regulation may contribute to T cell lymphomagenesis [20]. Although the clinical significance of *SETD2* mutations in primary CNS T cell lymphoma has not been fully revealed, the presence of a *SETD2* mutation in our case suggests a potential role in disease pathogenesis and aggressive clinical course.

This molecular finding suggests that comprehensive genomic profiling may be valuable for risk stratification and for the development of molecularly informed therapeutic strategies in primary CNS T cell lymphoma.

In conclusion, the case of a 37-year-old man with primary CNS T cell lymphoma involving both the brain parenchyma and spinal cord illustrates the aggressive clinical course and limited therapeutic options of this rare entity. The findings highlight the need for further research to develop more effective treatment strategies and improve outcomes for patients with primary CNS T cell lymphoma.

## New findings

We report an extremely rare case of primary central nervous system T cell lymphoma with simultaneous involvement of the brain parenchyma and extensive spinal cord lesions. Comprehensive molecular profiling identified a SETD2 mutation with a high variant allele frequency. Despite an initial radiological complete response to high-dose methotrexate-based therapy, the disease showed rapid and aggressive CNS relapse. This case highlights the potential role of molecular alterations in driving aggressive clinical behavior and underscores the importance of genomic analysis in this rare entity.

## Supplementary Information

Below is the link to the electronic supplementary material.

Supplementary file1 (DOCX 610 KB)

## Data Availability

The data supporting the findings of this study are available from the corresponding author upon reasonable request.The data are not publicly available due to patient privacy and ethical restrictions.

## References

[1] Camilleri-Broët S, Martin A, Moreau A, Angonin R, Hénin D, Gontier MF, et al. Primary central nervous system lymphomas in 72 immunocompetent patients: pathologic findings and clinical correlations. Groupe Ouest Est d’étude des Leucénies et Autres Maladies du Sang (GOELAMS). Am J Clin Pathol. 1998;110:607–12. 10.1093/ajcp/110.5.607 9802345

[2] Ferreri AJ, Blay JY, Reni M, Pasini F, Spina M, Ambrosetti A, et al. Prognostic scoring system for primary CNS lymphomas: the International Extranodal Lymphoma Study Group experience. J Clin Oncol. 2003;21:266–72. 10.1200/JCO.2003.09.139 12525518

[3] Chihara D, Fowler NH, Oki Y, Fanale MA, Nastoupil LJ, Westin JR, et al. Impact of histologic subtypes and treatment modality among patients with primary central nervous system lymphoma: a SEER database analysis. Oncotarget. 2018;9:28897–902. 10.18632/oncotarget.25622 PMC6034756 29988979

[4] Shenkier TN, Blay JY, O’Neill BP, Poortmans P, Thiel E, Jahnke K, et al. Primary CNS lymphoma of T-cell origin: a descriptive analysis from the international primary CNS lymphoma collaborative group. J Clin Oncol. 2005;23:2233–9. 10.1200/JCO.2005.07.109 15800313

[5] Scott BJ, Douglas VC, Tihan T, Rubenstein JL, Josephson SA. A systematic approach to the diagnosis of suspected central nervous system lymphoma. JAMA Neurol. 2013;70:311–9. 10.1001/jamaneurol.2013.606 PMC4135394 23319132

[6] Takahashi T, Sawada K, Yamashita T, Yamamoto W, Iijima Y, Adachi A, et al. Genetic profiling reveals the distinctions among MTX-associated DLBCL, EBV-positive Mucocutaneous Ulcer, and EBV + DLBCL. Cancer Sci. 2025;116:2306–16. 10.1111/cas.70111 PMC12317404 40458922

[7] Ito K, Imada H, Nagata M, Yamashita T, Sawada K, Takahashi T, et al. Significance and Utility of Immunohistochemical MTAP Expression to Predict CDKN2A Genomic Status in Diffuse Large B-Cell Lymphoma. Pathol Int. 2025. 10.1111/pin.70072 41410267

[8] van Dongen JJ, Langerak AW, Brüggemann M, Evans PA, Hummel M, Lavender FL, et al. Design and standardization of PCR primers and protocols for detection of clonal immunoglobulin and T-cell receptor gene recombinations in suspect lymphoproliferations: report of the BIOMED-2 Concerted Action BMH4-CT98-3936. Leukemia. 2003;17:2257–317. 10.1038/sj.leu.2403202 14671650

[9] Schaff LR, Grommes C. Primary central nervous system lymphoma. Blood. 2022;140:971–9. 10.1182/blood.2020008377 PMC9437714 34699590

[10] Guzzetta M, Drexler S, Buonocore B, Donovan V. Primary CNS T-cell lymphoma of the spinal cord: case report and literature review. Lab Med. 2015;46:159–63. 10.1309/LM85GPXDT8NJSDAB 25918197

[11] Lee DK, Chung CK, Kim HJ, Kim K, Choe G, Moon CW, et al. Multifocal primary CNS T cell lymphoma of the spinal cord. Clin Neuropathol. 2002;21:149–55. 12143926

[12] Levin N, Soffer D, Grissaru S, Aizikovich N, Gomori JM, Siegal T. Primary T-cell CNS lymphoma presenting with leptomeningeal spread and neurolymphomatosis. J Neurooncol. 2008;90:77–83. 10.1007/s11060-008-9633-2 18592137

[13] Hiroshima Y, Kaiume H, Kirihara T, Takeda W, Kurihara T, Sato K, et al. Successful long-term control of recurrent primary central nervous system anaplastic large cell lymphoma after autologous hematopoietic stem cell transplantation with concurrent whole brain and spinal cord radiotherapy. Rinsho Ketsueki. 2015;56:2477–82. 10.11406/rinketsu.56.2477 26725359

[14] Flanagan EP, O’Neill BP, Porter AB, Lanzino G, Haberman TM, Keegan BM. Primary intramedullary spinal cord lymphoma. Neurology. 2011;77:784–91. 10.1212/WNL.0b013e31822b00b9 21832220

[15] O’Brien PC, Roos DE, Pratt G, Liew KH, Barton MB, Poulsen MG, et al. Combined-modality therapy for primary central nervous system lymphoma: long-term data from a Phase II multicenter study (Trans-Tasman Radiation Oncology Group). Int J Radiat Oncol Biol Phys. 2006;64:408–13. 10.1016/j.ijrobp.2005.07.958 16198065

[16] Morris PG, Correa DD, Yahalom J, Raizer JJ, Schiff D, Grant B, et al. Rituximab, methotrexate, procarbazine, and vincristine followed by consolidation reduced-dose whole-brain radiotherapy and cytarabine in newly diagnosed primary CNS lymphoma: final results and long-term outcome. J Clin Oncol. 2013;31:3971–9. 10.1200/JCO.2013.50.4910 PMC5569679 24101038

[17] Menon MP, Nicolae A, Meeker H, Raffeld M, Xi L, Jegalian AG, et al. Primary CNS T-cell lymphomas: a clinical, morphologic, immunophenotypic, and molecular analysis. Am J Surg Pathol. 2015;39:1719–29. 10.1097/PAS.0000000000000503 PMC4644095 26379152

[18] Yim J, Koh J, Kim S, Song SG, Bae JM, Yun H, et al. Clinicopathologic and genetic features of primary T-cell lymphomas of the central nervous system: an analysis of 11 cases using targeted gene sequencing. Am J Surg Pathol. 2022;46:486–97. 10.1097/PAS.0000000000001859 PMC8923358 34980830

[19] Roberti A, Dobay MP, Bisig B, Vallois D, Boéchat C, Lanitis E, et al. Type II enteropathy-associated T-cell lymphoma features a unique genomic profile with highly recurrent *SETD2* alterations. Nat Commun. 2016;7:12602. 10.1038/ncomms12602 PMC5023950 27600764

[20] Moffitt AB, Ondrejka SL, McKinney M, Rempel RE, Goodlad JR, Teh CH, et al. Enteropathy-associated T cell lymphoma subtypes are characterized by loss of function of SETD2. J Exp Med. 2017;214:1371–86. 10.1084/jem.20160894 PMC5413324 28424246

