# Supplementary material for: Primary central nervous system T cell lymphoma involving multiple brain and spinal cord lesions with SETD2 mutation
Source: Int J Hematol. 2026 Aug 4;124(4):594–8. doi: 10.1007/s12185-026-04262-7 (PMC13638700; doi:10.1007/s12185-026-04262-7)
Supplement: Supplementary file 1 [file 12185_2026_4262_MOESM1_ESM.docx]

**Supplemental Figure 1:** **TCR rearrangement analysis by PCR according to the BIOMED-2 protocol (Leukemia. 2003; 17:2257-2317). Clonal rearrangements of TCRβ (tube B) and TCRγ (tubes A and B) were detected in the present case. Jurkat and SUDHL1 cell lines served as positive controls. Molecular size markers are shown on the left side of each image. N, negative control.**
